# Outcome Discrepancies and Selective Reporting: Impacting the Leading Journals?

**DOI:** 10.1371/journal.pone.0127495

**Published:** 2015-05-21

**Authors:** Padhraig S. Fleming, Despina Koletsi, Kerry Dwan, Nikolaos Pandis

**Affiliations:** 1 Oral Growth and Development, Barts and The London School of Medicine and Dentistry, Institute of Dentistry, Queen Mary University of London, London, England; 2 Department of Orthodontics, University of Athens, Athens, Greece; 3 Institute of Translational Medicine, University of Liverpool, Liverpool, England; 4 Department of Orthodontics and Dentofacial Orthopedics, Dental School/Medical Faculty, University of Bern, Bern, Switzerland; University Paris Descartes, FRANCE

## Abstract

**Background:**

Selective outcome reporting of either interesting or positive research findings is problematic, running the risk of poorly-informed treatment decisions. We aimed to assess the extent of outcome and other discrepancies and possible selective reporting between registry entries and published reports among leading medical journals.

**Methods:**

Randomized controlled trials published over a 6-month period from July to December 31^st^, 2013, were identified in five high impact medical journals: The *Lancet*, *British Medical Journal*, *New England Journal of Medicine*, *Annals of Internal Medicine* and *Journal of American Medical Association* were obtained. Discrepancies between published studies and registry entries were identified and related to factors including registration timing, source of funding and presence of statistically significant results.

**Results:**

Over the 6-month period, 137 RCTs were found. Of these, 18% (n = 25) had discrepancies related to primary outcomes with the primary outcome changed in 15% (n = 20). Moreover, differences relating to non-primary outcomes were found in 64% (n = 87) with both omission of pre-specified non-primary outcomes (39%) and introduction of new non-primary outcomes (44%) common. No relationship between primary or non-primary outcome change and registration timing (prospective or retrospective; P = 0.11), source of funding (P = 0.92) and presence of statistically significant results (P = 0.92) was found.

**Conclusions:**

Discrepancies between registry entries and published articles for primary and non-primary outcomes were common among trials published in leading medical journals. Novel approaches are required to address this problem.

## Introduction

The spotlight has been placed on deficient conduct and reporting of research in recent years [1]. In view of the profound influence of research findings on public health policy, the configuration of services and the delivery of care, these limitations are concerning [2]. The issue of publication bias, whereby positive or interesting results are more likely to be published and may be given greater prominence and priority than negative results is a significant problem [3]. Discrepancy between pre-specified and published outcomes and key methodological aspects of clinical trials is also prevalent and potentially problematic [4]. Inconsistencies between outcomes, in particular, may lead to biased and misleading results, particularly if these changes are predicated on the observed trial results. Selective reporting may manifest as preferential publication of either interesting or positive research findings, while less interesting, often negative, results are not published or their importance is downgraded. Moreover, only results from certain time points may be presented [4].

Reporting discrepancies and selective outcome reporting may lead to incorrect inferences, which in turn may culminate in uninformed and inappropriate treatment choices. The CONSORT (Consolidated Standards of Reporting Trials) guidelines are directed at promoting clear and unbiased reporting of clinical trials [5]. Within these guidelines it has been suggested that primary and non-primary outcomes should be defined in a clear manner in conjunction with effect sizes and confidence intervals [5]. *Post hoc* adjustments and subgroup analyses should be clear so biased or data-driven changes can be exposed. There is also empirical evidence both of inconsistencies and selective outcome reporting in both medical and surgical journals with issues exposed both in relation to primary and non-primary outcome reporting [6–11].

Registration of clinical trials has been advocated to introduce greater clarity. Clinical trial registries can be analysed to look for differences between the original entry study and the final article. This process can both help to identify selective outcome reporting and to find other inconsistencies. Mandatory trial registration has been adopted widely with the International Committee of Medical Journal Editors advocating registration prior to consideration for publication in a member journal [12]. The World Health Organization (WHO) has subsequently published a Trial Registration Data Set with 20 core items, including the primary and key non-primary outcomes [13].

Previous studies have highlighted a higher prevalence of methodological problems among both randomised controlled trials (RCTs) and systematic reviews within medical journals with a lower impact factor [14, 15]. With the exception of an analysis of reporting within the Journal of the American Medical Association and British Medical Journal [16], there is no evidence relating to the prevalence of discrepancies between registry entries and final reports within the leading medical journals. The aim of this study was to identify the prevalence of discrepancies between trial registry entries and final reports of RCTs published in leading medical journals with the highest impact factor. The differences between pre-specified and final outcome measures were to be determined. Alterations to be considered included addition or omission of outcome measures, changes from primary to non-primary outcomes or *vice versa*, and changes in the definition of outcome measures. Associations between discrepancies and a range of factors including statistical significance and funding sources was also to be explored.

## Materials and Methods

Randomised controlled trials published over a 6-month period from July to December 31^st^, 2013 were identified in five highest impact medical journals based on Thomson Reuters List 2014 by searching the electronic archives of the following: The *Lancet*, *British Medical Journal (BMJ)*, *New England Journal of Medicine (NEJM)*, *Annals of Internal Medicine (AIM)* and *Journal of American Medical Association (JAMA)*. Two authors (PSF, DK) identified RCTs for inclusion by independent searching of the electronic archives of these journals. Associated registry entries were identified within the abstract or methods section of the published manuscript.

Data from eligible studies were extracted independently and entered on pre-piloted standardized forms. Initial calibration was performed between the two researchers (PSF, DK) on 10 articles. Disagreements were resolved by discussion or, if necessary, with adjudication by a third researcher (NP). Information obtained included number and type of primary and non-primary outcomes based on explicit definitions within both the registry entries and the final publication. Changes in outcomes including addition, removal or redefinition of outcomes were recorded. An example of definition change would be a registry indicating, in a hypothetical study of a hypoglycemic agent, that the primary outcome was glycemic control assessed at 1 month, while glycemic control assessed at 2 months was reported in the study. However, if the registry entry reported the outcome without the time-point and the time-point was added in the report, this was noted but was not recorded as a discrepancy overall as additional detail is typical within trial reports.

In addition, data relating to changes to selection criteria, randomization and blinding procedures, analysis (per-protocol or intention-to-treat), and timing of assessments, sample size, funding, ethics and sponsorship were obtained. Estimates and associated confidence intervals where available were extracted for the primary outcome. Other characteristics including the trial register used and whether the research involved a single or multiple centers were also extracted.

Descriptive statistics were calculated for all included studies for the following variables: journal of publication, number of research centers, significance of the primary outcome, and registry used. Data were collected in relation to possible discrepancies between registry entries and final reports both for primary and non-primary outcomes. Other discrepancies in relation to trial design, randomization procedures, blinding, approach to analysis (intention to treat or per-protocol), incorporation of subgroup analyses, sample size, funding and registration details, timing of registration (retrospective or prospective), and allocation ratio were identified. Cross-tabulations were undertaken to investigate associations between discrepancies and timing of registration, type of sponsorship, and presence of significant results. All statistical analyses were conducted with STATA version 12.1 software (Stata Corporation, College Station, Texas, USA).

## Results

**Fig 1:**
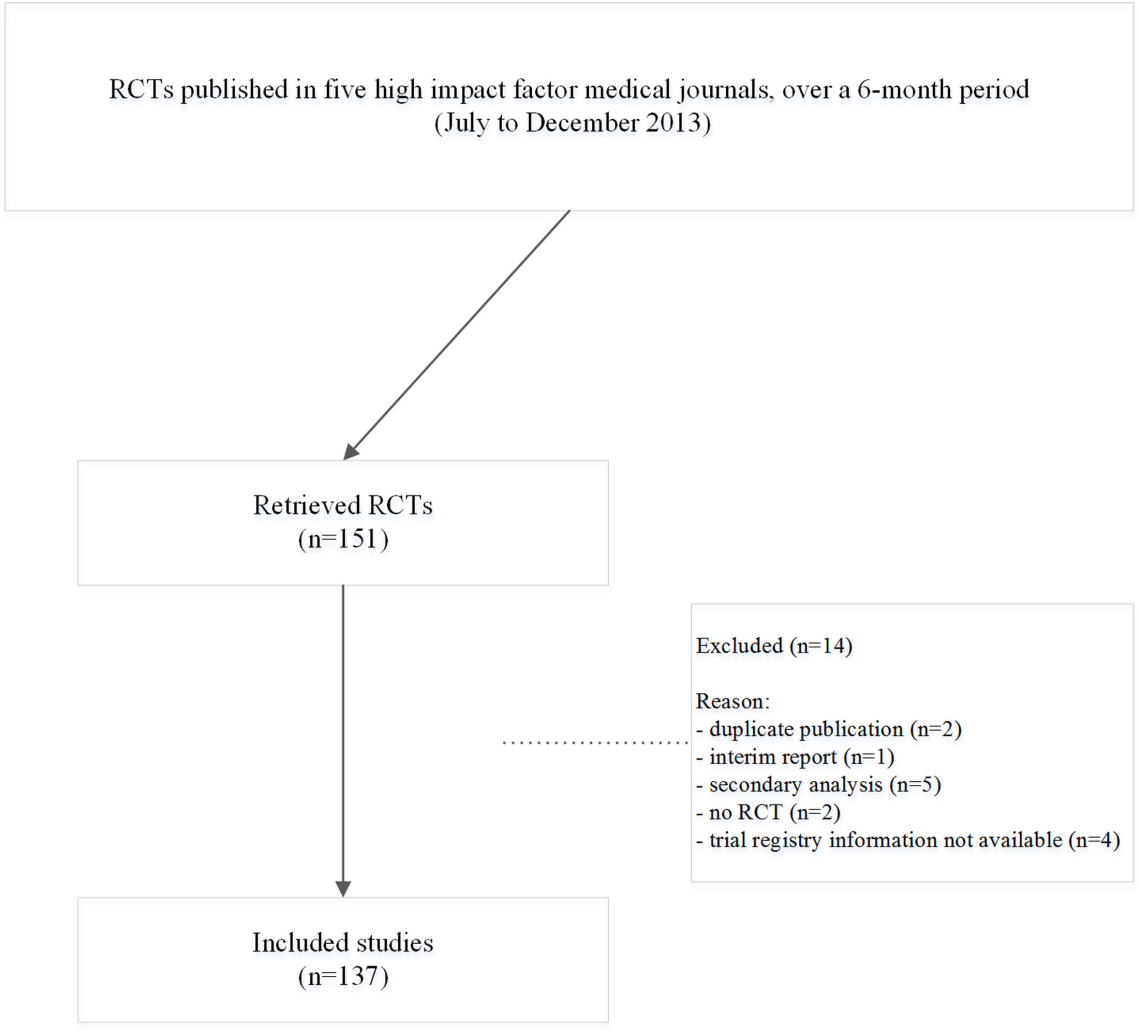
Flow diagram for article retrieval.

One hundred and thirty-seven RCTs were identified across the five journals (Fig 1, S1 Table). The greatest proportion of RCTs were identified in *NEJM* (n = 47, 34%), followed by *Lancet* (n = 32, 23%) and *JAMA* (n = 31, 22%). The vast majority were undertaken in multiple centers (n = 124, 91%), with a relatively even spread between significant (n = 67, 49%), and non-significant primary outcomes (n = 70, 51%). Of the various trial registries clinicaltrials.gov (n = 98, 72%) and controlled-trials.com (n = 25, 18%) were most popular. Most primary outcomes were dichotomous (n = 91, 66%).

Discrepancies related to primary outcomes were identified in 18% (n = 25) of the RCTs assessed. The definition of the primary outcome had changed in 15% (n = 20). In relation to non-primary outcomes, differences were found for 64% (n = 87) with similar frequency of both omission of pre-specified non-primary outcomes (39%) and introduction of new non-primary outcomes (44%) in published studies. No discrepancies related to either the intervention or design of the trial were identified. However, inclusion and exclusion criteria were altered in 24% (n = 33). Randomization procedures and approach to analysis were rarely outlined in the registries (96%). Subgroup analyses were described in the final report but not in the registry commonly (54%, n = 74). Certain discrepancies were found more commonly in individual journals including changes relating to randomization, blinding, subgroup analyses, funding and sponsorship (Table 1).

**Table 1:** Distribution of discrepancy variables between registration and published report per journal (n = 137).

Variables	Journal						
	AIM	BMJ	JAMA	Lancet	NEJM	Total	p-value
	N (%)	N (%)	N (%)	N (%)	N (%)	N (%)	
**Significance of primary outcome**							0.17^≠^
*Non-significant*	2 (20.0)	12 (70.6)	16 (51.6)	16 (50.0)	24 (51.1)	70 (51.1)	
*Significant*	8 (80.0)	5 (29.4)	15 (48.4)	16 (50.0)	23 (48.9)	67 (48.9)	
**Timing of registration**							0.20^≠^
*Prospective*	10 (100.0)	15 (88.2)	30 (96.8)	29 (90.6)	38 (80.8)	122 (89.1)	
*Retrospective*	0 (0.0)	2 (11.8)	1 (3.2)	3 (9.4)	9 (19.2)	15 (10.9)	
**Discrepancy in primary outcome**							0.25^≠^
*No*	10 (100.0)	11 (64.7)	26 (83.9)	27 (84.4)	38 (80.8)	112 (81.7)	
*Yes*	0 (0.0)	6 (35.3)	5 (16.1)	5 (15.6)	9 (19.2)	25 (18.3)	
**Discrepancy in non-primary outcome**							0.14^≠^
No	7 (70.0)	7 (41.2)	10 (32.3)	8 (25)	18 (38.3)	50 (36.5)	
Yes	3 (30.0)	10 (58.8)	21 (67.7)	24 (75)	29 (61.7)	87 (63.5)	
**Discrepancy in inclusion criteria**							0.27^≠^
*No*	10 (100.0)	13 (76.5)	26 (83.9)	22 (68.7)	32 (68.1)	103 (75.2)	
*Yes*	0 (0.0)	4 (23.5)	5 (16.1)	10 (31.3)	14 (29.8)	33 (24.1)	
*Not reported*	0 (0.0)	0 (0.0)	0 (0.0)	0 (0.0)	1 (2.1)	1 (0.7)	
**Discrepancy in randomization**							0.005^≠^
*No*	0 (0.0)	3 (17.6)	1 (3.2)	5 (15.6)	0 (0.0)	9 (6.6)	
*Yes*	0 (0.0)	3 (17.6)	1 (3.2)	1 (3.1)	1 (2.1)	6 (4.3)	
*Stated in registry*, *not in final report*	0 (0.0)	1 (5.9)	1 (3.2)	0 (0.0)	0 (0.0)	2 (1.5)	
*Stated in final report*, *not in registry*	10 (100.0)	10 (58.9)	26 (83.9)	26 (81.3)	45 (95.8)	117 (85.4)	
*Stated neither in registry*, *nor in final report*	0 (0.0)	0 (0.0)	2 (6.5)	0 (0.0)	1 (2.1)	3 (2.2)	
**Discrepancy in blinding**							<0.001^≠^
*No*	9 (90.0)	8 (47.1)	24 (77.4)	20 (62.5)	44 (93.6)	105 (76.6)	
*Yes*	1 (10.0)	1 (5.8)	6 (19.4)	3 (9.4)	0 (0.0)	11 (8.1)	
*Stated in registry*, *not in final report*	0 (0.0)	0 (0.0)	1 (3.2)	0 (0.0)	0 (0.0)	1 (0.7)	
*Stated in final report*, *not in registry*	0 (0.0)	8 (47.1)	0 (0.0)	9 (28.1)	2 (4.3)	19 (13.9)	
*Stated neither in registry*, *nor in final report*	0 (0.0)	0 (0.0)	0 (0.0)	0 (0.0)	1 (2.1)	1 (0.7)	
**Discrepancy in population analysis**							0.16^≠^
No	0 (0.0)	1 (5.8)	2 (6.5)	2 (6.3)	0 (0.0)	5 (3.7)	
Stated in final report, not in registry	8 (80.0)	13 (76.5)	28 (90.3)	29 (90.6)	42 (89.4)	120 (87.6)	
*Stated neither in registry*, *nor in final report*	2 (20.0)	3 (17.7)	1 (3.2)	1 (3.1)	5 (10.6)	12 (8.7)	
**Discrepancy in outcome time-points**							0.07^≠^
*No*	10 (0.0)	11 (64.7)	28 (90.3)	27 (84.4)	32 (68.1)	108 (78.8)	
*Yes*	0 (0.0)	6 (35.3)	3 (9.7)	5 (15.6)	13 (27.7)	27 (19.7)	
*Stated in registry*, *not in final report*	0 (0.0)	0 (0.0)	0 (0.0)	0 (0.0)	2 (4.2)	2 (1.5)	
**Discrepancy in subgroup analysis**							0.005^≠^
*No*	0 (0.0)	0 (0.0)	2 (6.5)	1 (3.1)	1 (2.1)	4 (2.9)	
*Yes*	0 (0.0)	4 (23.5)	0 (0.0)	0 (0.0)	0 (0.0)	4 (2.9)	
*Stated in final report*, *not in registry*	4 (40.0)	4 (23.5)	19 (61.3)	16 (50.0)	31 (65.9)	74 (54)	
*Stated neither in registry*, *nor in final report*	6 (60.0)	9 (53.0)	10 (32.2)	15 (46.9)	15 (32)	55 (40.2)	
**Discrepancy in sample size**							0.58^≠^
*No*	5 (50.0)	9 (52.9)	16 (51.6)	21 (65.6)	23 (48.9)	74 (54)	
*Yes*	5 (50.0)	8 (47.1)	15 (48.4)	10 (31.3)	24 (51.1)	62 (45.3)	
*Stated in registry*, *not in final report*	0 (0.0)	0 (0.0)	0 (0.0)	1 (3.1)	0 (0.0)	1 (0.7)	
**Discrepancy in funding**							0.004^≠^
*No*	8 (80.0)	15 (88.2)	17 (54.8)	26 (81.3)	44 (93.6)	110 (80.3)	
*Yes*	0 (0.0)	0 (0.0)	2 (6.5)	1 (3.1)	1 (2.1)	4 (2.9)	
*Stated in final report*, *not in registry*	2 (20.0)	2 (11.8)	12 (38.7)	5 (15.6)	2 (4.3)	23 (16.8)	
**Discrepancy in sponsorship**							0.002^≠^
No	9 (90.0)	13 (76.5)	23 (74.2)	18 (56.3)	25 (53.2)	88 (64.2)	
*Yes*	0 (0.0)	0 (0.0)	5 (16.1)	1 (3.1)	0 (0.0)	6 (4.4)	
*Stated in registry*, *not in final report*	1 (10.0)	4 (23.5)	3 (9.7)	13 (40.6)	21 (44.7)	42 (30.7)	
*Stated neither in registry*, *nor in final report*	0 (0.0)	0 (0.0)	0 (0.0)	0 (0.0)	1 (2.1)	1 (0.7)	
**Total**	10 (100.0)	17 (100.0)	31 (100.0)	32 (100.0)	47 (100.0)	137 (100.0)	

^≠^Fisher’s exact test

Regarding primary outcomes, no association was found for outcome change (report vs registry entry) or omission in the final report and registration timing (prospective or retrospective; P = 0.11), source of funding (P = 0.92) and presence of statistically significant results (P = 0.92). Similarly, no association was observed for these variables concerning changes in non-primary outcomes or where the primary outcome was omitted from the final publication (Table 2). An association between change in inclusion criteria and source of funding was noted with company-sponsored trials more likely to show changes (P = 0.014). No other statistically significant findings were observed.

**Table 2:** Associations between discrepancies and timing of registration, type of sponsorship, and presence of significant results.

	Discrepancy between registry and publication for the primary outcome				p-value
	Yes	No	Not reported	Total	
	N (%)	N (%)	N (%)	N (%)	
**Timing of registration**					0.11*
*Prospective*	20 (80.0)	102 (91.1)	-	122 (89.0)	
*Retrospective*	5 (20.0)	10 (8.9)	-	15 (11.0)	
**Funding Source**					0.92^≠^
*Government/university*	17 (68.0)	68 (60.7)	-	85 (62.0)	
*Company/corporation*	8 (32.0)	40 (35.7)	-	48 (35.0)	
*None/not reported*	0 (0.0)	1 (0.9)	-	1 (0.7)	
*Both*	0 (0.0)	3 (2.7)	-	3 (2.3)	
**Significance of Primary Outcome**					0.92*
*Non-significant*	13 (52.0)	57 (50.9)	-	70 (51.1)	
*Significant*	12 (48.0)	55 (49.1)	-	67 (48.9)	
**Total**	25 (100.0)	112 (100.0)	-	137 (100.0)	
	**Discrepancy between registry and publication for the non-primary outcome**				
**Timing of registration**					0.39*
*Prospective*	79 (90.8)	43 (86.0)	-	122 (89.1)	
*Retrospective*	8 (9.2)	7 (14.0)	-	15 (10.9)	
**Funding Source**					0.20^≠^
*Government/university*	51 (58.6)	34 (68.0)	-	85 (62.0)	
*Company/corporation*	33 (37.9)	15 (30.0)	-	48 (35.0)	
*None/not reported*	0 (0.0)	1 (2.0)	-	1 (0.8)	
*Both*	3 (3.5)	0 (0.0)	-	3 (2.2)	
**Significance of Primary Outcome**					0.58*
*Non-significant*	46 (52.9)	24 (48.0)	-	70 (51.1)	
*Significant*	41 (47.1)	26 (52.0)	-	67 (48.9)	
**Total**	87 (100.0)	50 (100.0)		137 (100)	
	**Primary outcome in registry omitted from publication**				
**Timing of registration**					0.99^≠^
*Prospective*	8 (88.9)	114 (89.1)	-	122 (89.1)	
*Retrospective*	1 (11.1)	14 (10.9)	-	15 (10.9)	
**Funding Source**					0.79^≠^
*Government/university*	5 (55.6)	80 (62.5)	-	85 (62)	
*Company/corporation*	4 (44.4)	44 (34.4)	-	48 (35.1)	
*None/not reported*	0 (0.0)	1 (0.8)	-	1 (0.7)	
*Both*	0 (0.0)	3 (2.3)	-	3 (2.2)	
**Significance of Primary Outcome**					0.99^≠^
*Non-significant*	5 (55.6)	65 (50.8)	-	70 (51.1)	
*Significant*	4 (44.4)	63 (49.2)	-	67 (48.9)	
**Total**	9 (100.0)	128 (100.0)		100 (137)	
	**Non-primary outcome in registry omitted from publication**				
**Timing of registration**					0.54*
*Prospective*	47 (87.0)	75 (90.4)	-	122 (89.1)	
*Retrospective*	7 (13.0)	8 (9.6)	-	15 (10.9)	
**Funding Source**					0.53^≠^
*Government/university*	36 (66.7)	49 (59)	-	85 (62)	
*Company/corporation*	18 (33.3)	30 (36.2)	-	48 (35.1)	
*None/not reported*	0 (0.0)	1 (1.2)	-	1 (0.7)	
*Both*	0 (0.0)	3 (3.6)	-	3 (2.2)	
**Significance of Primary Outcome**					0.89*
*Non-significant*	28 (51.9)	42 (50.6)	-	70 (51.1)	
*Significant*	26 (48.1)	41 (49.4)	-	67 (48.9)	
**Total**	54 (100.0)	83 (100.0)		137 (100.0)	
	**Discrepancy between registry and publication for the inclusion criteria**				
**Timing of registration**					0.99^≠^
*Prospective*	30 (90.9)	91 (88.3)	1 (100.0)	122 (89.1)	
*Retrospective*	3 (9.1)	12 (11.7)	0 (0.0)	15 (10.9)	
**Funding Source**					0.014^≠^
*Government/university*	14 (42.4)	71 (68.9)	0 (0.0)	85 (62.1)	
*Company/corporation*	17 (51.5)	30 (29.1)	1 (100.0)	48 (35)	
*None/not reported*	0 (0.0)	1 (1.0)	0 (0.0)	1 (0.7)	
*Both*	2 (6.1)	1 (1.0)	0 (0.0)	3 (2.2)	
**Significance of Primary Outcome**					0.77^≠^
*Non-significant*	16 (48.9)	54 (52.4)	0 (0.0)	70 (51.1)	
*Significant*	17 (51.5)	49 (47.6)	1 (100.0)	67 (48.9)	
**Total**	33 (100.0)	103 (100.0)	1 (100.0)	137 (100.0)	

*chi-square,

^≠^Fisher’s exact test

## Discussion

The presence of inconsistency between clinical trial reports and registry entries has previously been confirmed in major medical and surgical journals and within drug trials [6–11]. This study confirms the pattern alluded to in these studies; however, an association between timing of registration, funding source and presence of significant outcomes and discrepancies relating to primary and non-primary outcomes was not found. Even in high impact journals, which all endorse trial registration in guidelines for authors, selective reporting remains problematic. A previous analysis of selective reporting within JAMA and BMJ highlighted a discrepancy rate of 19% and 47%, respectively [16]. Analogous rates of discrepancy were exposed in the present analysis suggesting that merely endorsing and promoting registration is not sufficient; further action is required to produce more meaningful improvements.

It appears that primary outcomes are being handled with greater fidelity than is the case with non-primary outcomes. Nevertheless, a sizeable proportion (18%) of studies were exposed as having discrepancies between primary outcomes on registry entries and in the final report. While this figure is considerably lower than that identified in a recent review of surgical studies, where rates of up to 49% were found [9], rates ranging from 18% to 43% have been found in previous studies [6–8, 10]. This problem stemmed relatively evenly from either omission of primary outcomes outlined on the registry, *de novo* introduction of previously unmentioned outcomes and downgrade of primary to non-primary outcomes. This distribution is in accordance with other studies reporting the previous two issues with a frequency of 8% to 18% [6, 8] and the latter at between 4% and 22% [6, 7]. This, therefore, confirms that trials published more recently in the most reputed journals are susceptible to analogous levels of selective reporting as was revealed up to 5 years ago [6–8].

In keeping with previous findings, the rate of disagreement between non-primary outcomes listed on registry entries and in final reports was 64%. The same figure was previously identified for surgical journals [6]; however, even higher levels (70%) have been exposed [7]. The most pervasive issues in the present report related to either omission (39%) or *de novo* introduction (44%) of non-primary outcomes in the published reports. Upgrade of non-primary outcomes to primary was relatively rare (2%); this figure corresponds with previous research alluding to a rate of 3% for this discrepancy [7], although levels of up to 14% have been reported [6]. No association was found between discrepancies in outcome reporting and statistical significance of the results. This finding may reflect a lack of power to identify a true difference. It may, however, be possible that outcomes are changed within clinical trials following genuine *post hoc* decisions, for example, based on pilot studies, recruitment or due to advice from data monitoring committees. Notwithstanding this, it is important that *post hoc* changes of this nature are delineated within the published manuscript, ideally accompanied by an explanation of the reasons for the change.

In respect of sample size, a discrepancy was found in 45% of trials. This figure is in general keeping with previous studies, which have alluded to differences in the region of 48% to 73% of entries [6, 17]. The veracity of the sample size calculations and the information contributing to the calculated values were not assessed in the present study; however, previous research has exposed problems in this respect [18, 19]. Consequently, further efforts are required both in relation to the clarity and conduct of sample size estimation in clinical trials. It is particularly important, however, that sample size estimation is delineated in trial reports due to the risk of biased *post hoc* decisions in an effort to demonstrate that clinical trials possessed the requisite power to demonstrate clinically relevant differences where they are identified [20].

A number of factors described in the final report were rarely delineated in the registry entry. Subgroup analyses were referred to in only 6% of registry entries, while analyses were reported in the final paper in 54% without previous mention in the register. Similarly, randomization procedures, ethical approval status (74%) and mode of population analysis (88%) were rarely outlined in registry entries. This pattern may be overcome by stipulating mandatory entries in relation to each of these areas on trial registers or by providing additional space to provide further relevant but non-essential information. It has been demonstrated that completion of mandatory components is almost universal on recognized databases (e.g. ClinicalTrials.gov) [21], while optional fields are completed with significantly lower frequency. Completion rates were just 66% and 56%, respectively, for primary and non-primary outcomes. While evidence pointing to improved outcomes favoring sponsors of published pharmaceutical studies and a recognized susceptibility to publication bias exists [22], in the present study no association between either primary or non-primary outcome change or omission and either registration timing or source of funding was found.

Despite the adoption, promotion and endorsement of reporting guidelines, there remain significant shortcomings [2]. The emphasis should, therefore, shift to implementing changes rather than allowing relatively passive submission, peer review and editorial consideration, while expecting improvements in reporting and conduct. While stipulation of mandatory elements on registry databases is effective, it may be possible to implement a registry modeled on CONSORT items completed prospectively to stimulate optimal trial design and clarity at the protocol stage. The Standard Protocol Items: Recommendations for Intervention (SPIRIT) checklist has recently been developed with the intention of clarifying the items required during protocol reporting. Within these 33 items relating to all aspects of trial design, including ethics and dissemination are mentioned [23]. If the SPIRIT guidelines become embraced as a standard technique for outlining protocols of clinical trials, it is likely that these omissions will be rectified, particularly if registry entries could be made to reflect more detailed protocols rather than being restricted to more limited fields. Biomedical journals are beginning to adopt the SPIRIT statement and as acceptance increases it is possible trial registries may be modeled more closely on SPIRIT guidance [24]. A further option might be to append the registry entry to final submissions for inspection during the peer review process with authors encouraged to outline discrepancies between them to promote greater clarity and candor.

Limitations of this study include its restriction to a six-month period and to a subset of just five journals. These journals were included as they are prominent, well regarded and have the highest impact factors. It was therefore hoped that these might represent best practice with more significant discrepancies arising in less prominent journals. While the latter appears to apply to methodological quality of both systematic reviews and RCTs, there is no conclusive evidence that this extends to selective outcome reporting [14, 15]. It would be intuitive to expect, however, that journals not stipulating the need for trial registration are likely to present similar or higher rates of discrepancy. Moreover, registry entries do not at this stage correspond to trial protocols as protocols are not usually publically available. It is possible, therefore, that information on registries was truncated deliberately in an effort to comply with registry policies. The benefit of encouraging more complete registry entries, with an onus on mandatory completion is, therefore, clear. Finally, it had been hoped to investigate the association between selective reporting and observed estimates; this was not possible due to the relatively low number of studies examined. It may be interesting, however, to investigate whether selective reporting of trial outcomes is associated with distortion of effect estimates likely resulting in a greater preponderance of positive outcomes for *de novo* primary or non-primary outcomes, which were not outlined in the registry.

## Conclusions

A high rate of discrepancies between registry entries and published articles for primary and non-primary outcomes were identified among a sample of trials published in leading medical journals. Selective outcome reporting, therefore, appears to be a persistent issue. Novel approaches are required to address this issue throughout biomedical research.

## Supporting Information

S1 Table Characteristics of the included RCTs (n = 137). (DOCX)
