# Supplementary material for: Outcome Discrepancies and Selective Reporting: Impacting the Leading Journals?
Source: PLoS One. 2015 May 21;10(5):e0127495. doi: 10.1371/journal.pone.0127495 (PMC4440809; doi:10.1371/journal.pone.0127495)
Supplement: S1 Table — (DOCX) [file pone.0127495.s001.docx]

**Appendix Table 1**. Characteristics of the included RCTs (n=137).

| **Characteristic** |  | **N** | **%** |
| --- | --- | --- | --- |
| **Journal** | *AIM* | 10 | 7 |
|  | *BMJ* | 17 | 12 |
|  | *JAMA* | 31 | 22 |
|  | *Lancet* | 32 | 23 |
|  | *NEJM* | 47 | 34 |
| **Number of centers** | *Single center* | 13 | 9 |
|  | *Multi center* | 124 | 91 |
| **Registry** | *clinicaltrials.gov* | 98 | 72 |
|  | *controlled-trials.com* | 25 | 18 |
|  | *Australian New Zealand Clinical Trials Registry* | 7 | 5 |
|  | *UMIN Clinical Trials Registry* | 1 | 1 |
|  | *Other* | 6 | 4 |
| **Type of primary outcome in registry** | *Binary/dichotomous* | 91 | 66 |
|  | *Continuous* | 46 | 34 |
| **Type of primary outcome in publication** | *Binary/dichotomous* | 91 | 66 |
|  | *Continuous* | 46 | 34 |
| **All primary outcome(s) stated in the trial registry is/are the same as in the published report** | *No* | 25 | 18 |
|  | *Yes* | 112 | 82 |
| **One or several primary outcome(s) stated in the trial registry is downgraded to non-primary in the published report** | *No* | 131 | 96 |
|  | *Yes* | 6 | 4 |
| **One or several primary outcome stated in the trial registry is/are omitted from the published report** | *No* | 128 | 93 |
|  | *Yes* | 9 | 7 |
| **One or several new primary outcome(s) that was/were not stated in the trial registry is included in the published report** | *No* | 131 | 96 |
|  | *Yes* | 6 | 4 |
| **The definition of one or several primary outcome(s) was different (although the same variable) in the trial registry compared to the published report** | *No* | 117 | 85 |
|  | *Yes* | 20 | 15 |
| **All non-primary outcomes stated in the trial registry are the same as in the published report** | *No* | 87 | 64 |
|  | *Yes* | 50 | 36 |
| **One or several non-primary outcome(s) in the trial registry is/are upgraded to primary in the published report** | *No* | 135 | 99 |
|  | *Yes* | 2 | 1 |
| **One or several non-primary outcome(s) stated in the trial registry is/are omitted from the published report** | *No* | 83 | 61 |
|  | *Yes* | 54 | 39 |
| **One or several new non-primary outcome(s) that was/were not stated in the trial registry is/are included in the published report** | *No* | 77 | 56 |
|  | *Yes* | 60 | 44 |
| **The definition of one or several non-primary outcome(s) was different (although the same variable) in the trial registry compared to the published report** | *No* | 132 | 96 |
|  | *Yes* | 5 | 4 |
| **Change in intervention** | *No* | 137 | 100 |
|  | *Yes* | 0 | 0 |
| **Change in inclusion criteria** | *No* | 103 | 75 |
|  | *Yes* | 33 | 24 |
|  | *Not reported* | 1 | 1 |
| **Change in exclusion criteria** | *No* | 102 | 74 |
|  | *Yes* | 33 | 24 |
|  | *Not reported* | 2 | 1 |
| **Trial design in registry** | *Parallel* | 117 | 86 |
|  | *Crossover* | 2 | 1 |
|  | *Factorial* | 7 | 5 |
|  | *Cluster* | 11 | 8 |
| **Trial design in publication** | *Parallel* | 117 | 86 |
|  | *Crossover* | 2 | 1 |
|  | *Factorial* | 7 | 5 |
|  | *Cluster* | 11 | 8 |
| **Population analysis publication** | *Per protocol* | 16 | 11 |
|  | *Intention to treat* | 97 | 71 |
|  | *Both* | 12 | 9 |
|  | *Not stated/ unclear* | 12 | 9 |
| **Funding type in publication** | *Government/University* | 85 | 62 |
|  | *Company/Corporation* | 48 | 35 |
|  | *None/not reported* | 1 | 1 |
|  | *Both* | 3 | 2 |
| **Sponsor type in publication** | *Government/University* | 65 | 48 |
|  | *Company/Corporation* | 28 | 20 |
|  | *None/not reported* | 43 | 31 |
|  | *Both* | 1 | 1 |
| **Change in ethical approval** | *No discrepancy* | 34 | 25 |
|  | *Discrepancy* | 1 | 1 |
|  | *Stated in registry, not in final report* | 0 | 0 |
|  | *Stated in final report, not in registry* | 102 | 74 |
|  | *Stated neither in registry, nor in final report* | 0 | 0 |
| **Number of Arms** | *2* | 114 | 83 |
|  | *3* | 15 | 11 |
|  | *4* | 6 | 4 |
|  | *>4* | 2 | 2 |
| **Total** |  | **137** | **100** |
